# Seeded assembly *in vitro* does not replicate the structures of α‐synuclein filaments from multiple system atrophy

**DOI:** 10.1002/2211-5463.13110

**Published:** 2021-02-24

**Authors:** Sofia Lövestam, Manuel Schweighauser, Tomoyasu Matsubara, Shigeo Murayama, Taisuke Tomita, Takashi Ando, Kazuko Hasegawa, Mari Yoshida, Airi Tarutani, Masato Hasegawa, Michel Goedert, Sjors H. W. Scheres

**Affiliations:** ^1^ MRC Laboratory of Molecular Biology Cambridge UK; ^2^ Department of Neuropathology Tokyo Metropolitan Institute of Gerontology Japan; ^3^ Graduate School of Pharmaceutical Sciences The University of Tokyo Japan; ^4^ Department of Neurology Nagoya University Graduate School of Medicine Japan; ^5^ Division of Neurology Sagamihara National Hospital Japan; ^6^ Institute for Medical Science of Aging Aichi Medical University Nagakute Japan; ^7^ Department of Brain and Neurosciences Tokyo Metropolitan Institute of Medical Science Japan

**Keywords:** alpha‐synuclein, amyloid, cryo electron microscopy, multiple system atrophy

## Abstract

The propagation of conformational strains by templated seeding is central to the prion concept. Seeded assembly of α‐synuclein into filaments is believed to underlie the prion‐like spreading of protein inclusions in a number of human neurodegenerative diseases, including Parkinson's disease, dementia with Lewy bodies (DLB) and multiple system atrophy (MSA). We previously determined the atomic structures of α‐synuclein filaments from the putamen of five individuals with MSA. Here, we used filament preparations from three of these brains for the *in vitro* seeded assembly of recombinant human α‐synuclein. We find that the structures of the seeded assemblies differ from those of the seeds, suggesting that additional, as yet unknown, factors play a role in the propagation of the seeds. Identification of these factors will be essential for understanding the prion‐like spreading of α‐synuclein proteinopathies.

## Graphical abstract

The assembly of certain proteins into amyloids underlies multiple neurodegenerative diseases. The spreading of these assemblies through the brain is thought to occur through a prion‐like mechanism. We used filaments extracted from multiple system atrophy brains to seed recombinant α‐synuclein. The resulting structures differ from those of the seeds, indicating that seeded assembly does not necessarily replicate the seed structures.

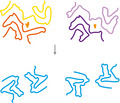

**Abbreviations**
AD: Alzheimer’s disease
Cryo‐EM: cryo electron microscopy
DLB: dementia with Lewy body
FSC: fourier shell correlation
GCI: glial cytoplasmic inclusions
MSA: multiple system atrophy
PBS: phosphate‐buffered saline
PD: Parkinson’s disease
PIPES: piperazine‐N, N′‐bis(2‐ethanesulfonic acid)
PMCA: protein misfolding cyclic amplification
RMSD: root‐mean‐square deviation
RT‐QuIC: real time‐induced quaking‐induced conversion
THT: thioflavin T

The ordered assembly of a small number of proteins into pathological amyloid filaments defines most neurodegenerative diseases, including Alzheimer disease (AD) and Parkinson disease (PD) [1]. Diseases characterized by the assembly of α‐synuclein and tau are the most common proteinopathies of the human nervous system. Most cases of disease are sporadic, but a small percentage is inherited.

The first assemblies form in a small number of cells in a given brain region, from where they spread through prion‐like mechanisms [1]. A central tenet of the prion hypothesis is that proteinopathies are characterized by assemblies with specific conformations that propagate from cell to cell [2, 3]. Spreading is consistent with staging schemes that have postulated a stereotypical progression of inclusions from single sites [4, 5]. Decades elapse between the formation of assemblies and the appearance of disease symptoms, providing an important therapeutic window. Evidence for the existence of prion‐like mechanisms in human brain has come from the development of scattered α‐synuclein inclusions in foetal human midbrain neurons that were therapeutically implanted into the striata of patients with advanced PD [6, 7].

α‐Synuclein assemblies are characteristic of PD, PD dementia, DLB, MSA and several rarer conditions, known collectively as synucleinopathies [8]. In these diseases, the 140 amino acid α‐synuclein assembles into a filamentous, β‐sheet‐rich conformation. Unbranched α‐synuclein filaments are 5–10 nanometres in diameter and up to several micrometres in length. They are found mostly in nerve cells (Lewy bodies and neurites) and, for MSA, also in glial cells, chiefly in oligodendrocytes (glial cytoplasmic inclusions, GCIs, or Papp‐Lantos bodies). Filamentous α‐synuclein is phosphorylated and exhibits additional post‐translational modifications [9, 10], but it remains to be shown that these modifications are necessary for assembly. Amino acids 30–100 have been reported to make up the structured part of α‐synuclein filaments [11]. A seed of α‐synuclein can trigger the assembly of soluble α‐synuclein [12].

A link between α‐synuclein assembly and disease was established by the findings that missense mutations in SNCA (the α‐synuclein gene), and multiplications of this gene, cause rare forms of inherited PD and PD dementia [13, 14]. Some SNCA mutations and gene multiplications also cause DLB. Abundant α‐synuclein inclusions are present in all cases of inherited disease. Sequence variation in the regulatory region of SNCA is associated with increased α‐synuclein expression and a heightened risk of developing sporadic PD, which accounts for over 90% of cases of this disease [15]. Expressed α‐synuclein, wild‐type or mutant, assembles into filaments *in vitro* [16]. Moreover, expression of human mutant α‐synuclein in animal models causes its aggregation and neurodegeneration [17].

Experimental evidence has shown that assembled α‐synuclein from MSA behaves like a prion [18]. Intracerebral or peripheral injection of MSA brain extracts into heterozygous mice transgenic for human A53T α‐synuclein led to the formation of abundant neuronal α‐synuclein inclusions and their spreading, accompanied by motor impairment [19, 20, 21, 22]. Protein misfolding cyclic amplification (PMCA) and real time‐induced quaking‐induced conversion (RT‐QuIC) have been reported to discriminate between MSA and PD [23].

Following the identification of α‐synuclein filaments from DLB by negative‐stain immuno‐electron microscopy (immuno‐EM) [24], multiple techniques, including solid‐state nuclear magnetic resonance, electron diffraction, X‐ray diffraction and electron cryo‐microscopy (cryo‐EM), have been used to study the molecular structures of recombinant α‐synuclein filaments [23, 25, 26, 27, 28, 29, 30, 31]. In some of these studies, filaments were also amplified by using seeds from human brain and recombinant human protein as substrate.

We recently showed that the structures of α‐synuclein filaments from MSA consist of type I and type II filaments, each with two different protofilaments [32]. By two‐dimensional class averaging, filaments from the brains of individuals with MSA differ from those of DLB, suggesting that distinct strains do indeed characterize synuclein proteinopathies. However, as is the case of tau assemblies [33], the structures of α‐synuclein filaments from brain are unlike those formed from recombinant proteins. The main differences are in the extended folds of MSA protofilaments, their asymmetrical packing and the presence of nonproteinaceous molecules between protofilaments.

These findings raised the question if seeded assemblies of α‐synuclein have the same structures as those of brain seeds. Here, we show that the cryo‐EM structures of seeded recombinant α‐synuclein assemblies do not necessarily replicate those of MSA seeds, suggesting that in disease, additional molecules and/or post‐translational modifications of α‐synuclein are required for the faithful replication of filament structures.

## Results

### Seeded assembly of α‐synuclein with filament preparations from MSA brains

**Fig. 1:**
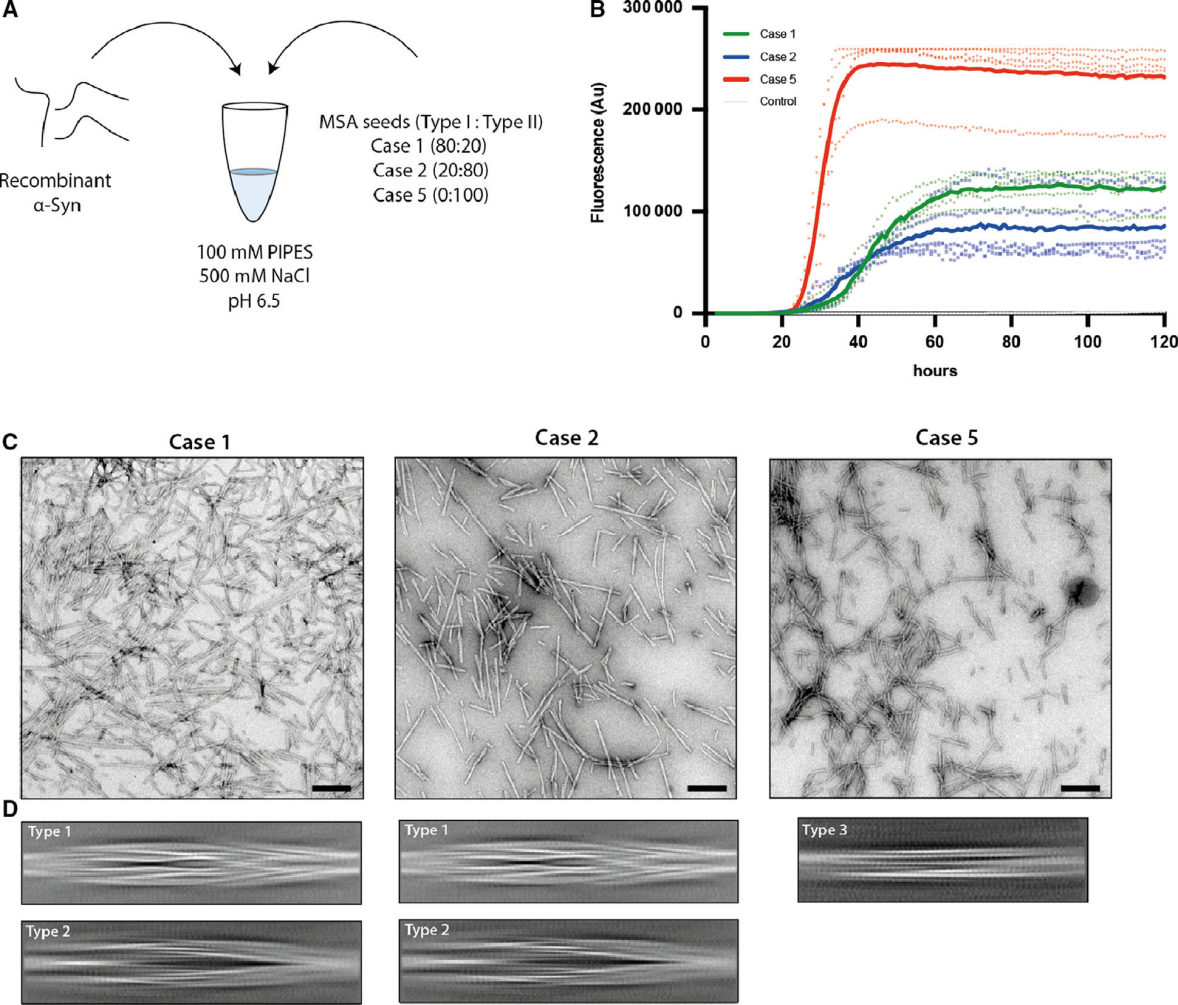
Seeded assembly of recombinant α‐synuclein with filament preparations from MSA brains. (A) Recombinant wild‐type human α‐synuclein was mixed with sonicated MSA seeds in 100 mm PIPES, 500 mm NaCl, 0.05% NaN_3_, pH 6.5. Seeds had variable ratios of type I and type II filaments. (B) Assembly was quantitated by thioflavin T fluorescence of recombinant α‐synuclein in the presence of MSA seeds from case 1 (green), case 2 (blue) and case 5 (red). Controls (grey) were without seeds. Curves represent the mean and dots correspond to the values in each experiment, (*n* = 5). (C) Negative stain micrographs of α‐synuclein filaments after seeded assembly (scale bar = 200 nm). (D) Cryo‐EM 2D class averages in boxes spanning 825 Å of the types of filaments. Assembly with seeds from MSA cases 1 and 2 gave rise to type 1 and type 2 filaments. Type 3 filaments formed when the seeds were from MSA case 5.

We seeded the *in vitro* assembly of recombinant wild‐type human α‐synuclein with filament preparations from the putamen of three cases of MSA (Materials and methods). The cryo‐EM structures of the filaments from these cases are known (cases 1, 2 and 5 in ref. [32]). They contain variable proportions of type I and type II MSA filaments, with I : II ratios of 80 : 20 for case 1; 20 : 80 for case 2; and 0 : 100 for case 5. We monitored the kinetics of aggregation using thioflavin T [34]. The assembly conditions were as described [23], using 100 mm piperazine‐N, N′‐bis(2‐ethanesulfonic acid) (PIPES) and 500 mm NaCl at 37 °C, pH 6.5. Upon addition of seeds, we observed a lag phase of 20–40 h, before fluorescence increased rapidly and plateaued after 30–60 h (Fig. 1B). Case 5 seeds were faster at seeding recombinant α‐synuclein and resulted in higher fluorescence intensities than seeds from cases 1 and 2. No increase in fluorescence was observed in the absence of seeds. Negative‐stain EM confirmed the presence of abundant filaments after incubation with MSA seeds (Fig. 1C).

### Cryo‐EM imaging of seeded α‐synuclein filaments

We used cryo‐EM to image the filaments formed following incubation of recombinant α‐synuclein with seeds from each MSA case. Visual inspection of micrographs of filaments from experiments that used seeds from MSA cases 1 and 2 indicated the presence of two main filament types, which we called type 1 and type 2. Type 1 filaments have an average cross‐over distance of 800 Å and widths of 60–130 Å; type 2 filaments have a cross‐over distance of 900 Å and widths of 80–130 Å. We also observed straight filaments with no observable twist. It is unclear if they correspond to filaments of types 1 or 2 that untwisted because of sample preparation artefacts, such as interactions with the air‐water interface, or if they represent additional filament types. Due to the lack of twist, we were unable to solve the structures of these filaments.

**Fig. 2:**
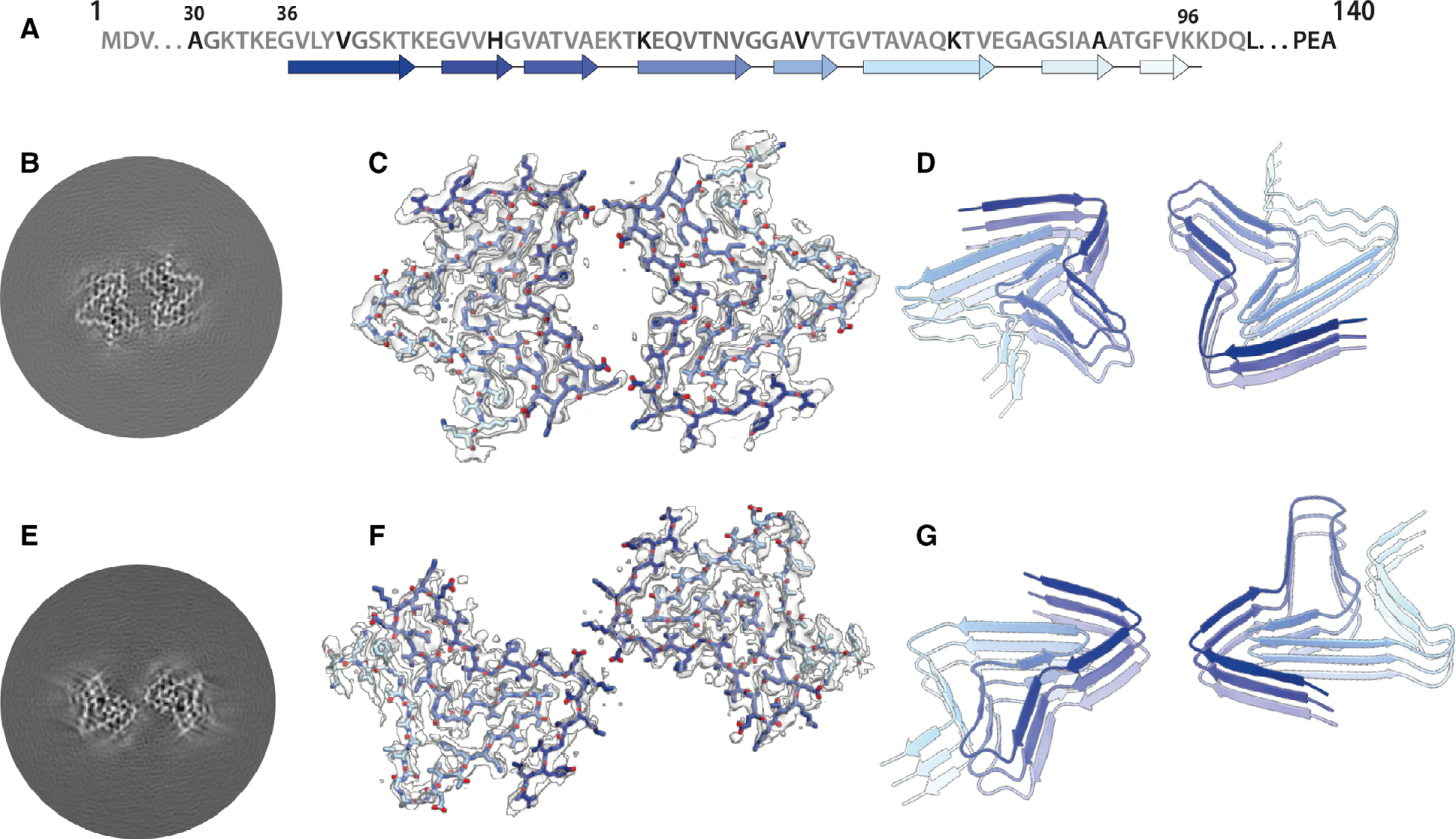
Cryo‐EM structures of type 1 and type 2 filaments with protofilament fold A assembled using seeds from MSA case 2. (A) Primary sequence of α‐synuclein with β‐strands and loop regions shown from dark blue (N‐terminal) to light blue (C‐terminal). (B) Central slice of the 3D map for type 1 filaments with protofilament fold A. (C) Cryo‐EM density (transparent grey) and fitted atomic model (with the same colour scheme as in a) for type 1 filaments. (D) Cartoon view of three successive rungs of the type 1 filament. (E––G) As (B–D), but for type 2 filaments.

**Fig. 3:**
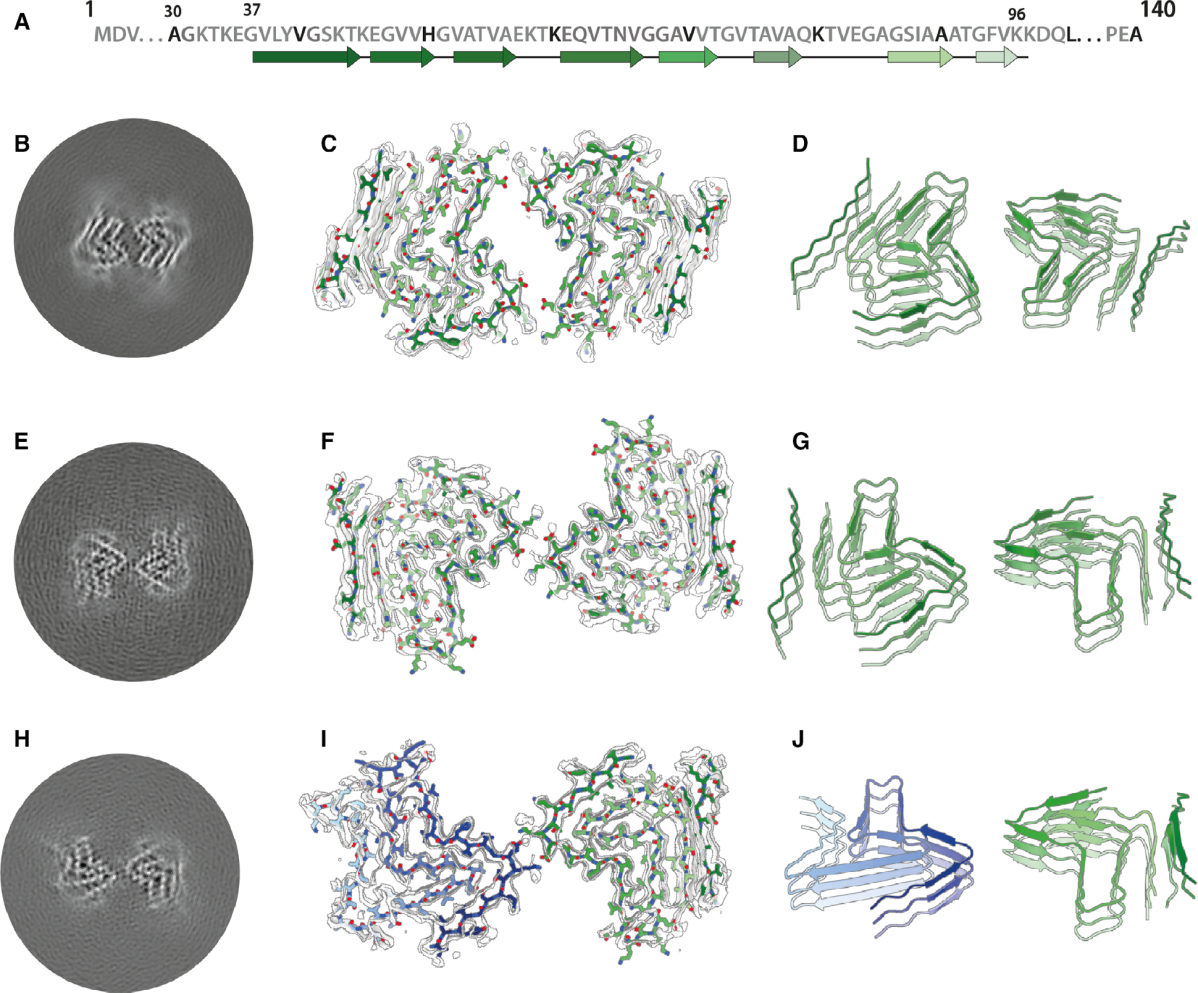
Cryo‐EM structures of type 1 and type 2 filaments with protofilament fold B assembled using seeds from MSA case 1. (A) Primary sequence of α‐synuclein with β‐strands and loop regions shown from dark green (N‐terminal) to light green (C‐terminal). (B) Central slice of the 3D map for type 1 filaments with protofilament fold B. (C) Cryo‐EM density (transparent grey) and fitted atomic model (with the same colour scheme as in A) for type 1 filaments. (D) Cartoon view of three successive rungs of the type 1 filament. (E–G) As (B–D), but for type 2 filaments. (H–I) As (B–D), but for the putative type 2 filament that contains a mixture of protofilament folds A and B.

**Fig. 4:**
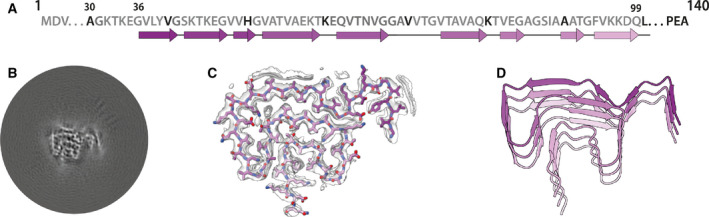
Cryo‐EM structure of type 3 filaments assembled using seeds from MSA case 5. (A) Primary sequence of α‐synuclein with β‐strands and loop regions shown from dark violet (N‐terminal) to light pink (C‐terminal). (B) Central slice of the 3D map for the type 3 filament. (C) Cryo‐EM density (transparent grey) and the fitted atomic model (with the same colour scheme as in A). (D) Cartoon view of three successive rungs of the type 3 filament.

Two‐dimensional classification readily separated type 1 and type 2 filaments for further processing and indicated that both types are 2‐fold symmetric along their helical axis (Fig. 1D). Further 3D classification revealed that type 1 and type 2 filaments occurred in two variants in the data set of filaments that formed with seeds from MSA case 1. They are characterized by small differences in protofilament folds. We called the predominant protofilament ‘fold A’ and the minor protofilament ‘fold B’. We could not identify protofilaments with fold B when seeds from MSA case 2 were used. Using helical reconstruction in RELION [35], we determined cryo‐EM structures of type 1 and type 2 filaments with only protofilament fold A to 3.4 Å resolution (Fig. 2; Figs S2 and S3). Reconstructions of type 1 and type 2 filaments with two protofilaments of fold B, or with one protofilament of fold A and another protofilament of fold B, were solved to resolutions of 3.4–4.1 Å (Fig. 3; Fig. S4). Reconstructions of filaments containing protofilaments of fold B were less well defined than those of filaments with two protofilaments of fold A. Assembly with seeds from MSA case 5 resulted almost exclusively in the formation of a different type of filament, which we called type 3. Type 3 filaments were thinner, more bendy and longer than filaments of types 1 and 2. Type 3 filaments have a crossover of 900 Å and widths of 55–65 Å. We solved their structure to 3.2 Å resolution (Fig. 4; Figs S8 and S9). A minority of filaments (< 2%) comprised a doublet of the type 3 filaments. Throughout this manuscript, we use blue colours for fold A and green for fold B of type 1 and type 2 filaments, and we use purple for type 3 filaments.

### Cryo‐EM structures of type 1 and type 2 α‐synuclein filaments

Most type 1 and type 2 filaments that formed with seeds from MSA case 1, and all the filaments that formed with seeds from MSA case 2, consisted of two protofilaments of fold A that were related by C2 symmetry. Filaments of types 1 and 2 differed in their inter‐protofilament packing (Fig. 2; Fig. S5). In type 1 filaments, two salt bridges between E46 and K58 held the protofilaments together, by creating a large solvent‐filled channel. The inter‐protofilament interface in type 2 filaments was formed by two salt bridges between K45 and E46 of each protofilament. The smeared reconstructed densities at the points furthest away from the helical axis suggest that the inter‐protofilament interface of type 2 filaments is more flexible than that of type 1 filaments. Protofilament fold A consists of 8 β‐sheets: β1‐6 form a roughly Z‐shaped hairpin‐like structure, with glycines or KTK motifs between the β‐sheets at the bends; β7‐8 fold back against β4, leaving a small triangular cavity between β5, β6 and β7. This fold is unlike any of those of the MSA type I and type II protofilaments. It is almost identical to the protofilament fold that was reported for *in vitro* aggregated recombinant E46K α‐synuclein [36], although the inter‐protofilament interface was different from the interfaces observed here for type 1 and type 2 filaments (Fig. 2; Fig. S4). A minority of type 1 and 2 filaments that formed with seeds from MSA case 1 consisted of two symmetry‐related copies of protofilaments with fold B. Although the reconstructions of type 1 and type 2 filaments with two protofilaments of fold B (Fig. 3; Fig. S4) were less well defined than those for filaments with two protofilaments of fold A, the maps revealed that fold B is nearly identical with the structure of filaments assembled from wild‐type recombinant α‐synuclein [30]. This increased our confidence in building and refining an atomic model for the protofilaments with fold B. The resulting model from the type 2 filament has a root‐mean‐square deviation (r.m.s.d.) of 1.38 Å with the structure of assembled wild‐type α‐synuclein (Fig. S6) [30]. Again, protofilament fold B was unlike any of the four protofilaments from MSA type I and type II filaments. An asymmetric reconstruction from a subset of the images suggested that asymmetric type 2 filaments may also form from one protofilament with fold A and one protofilament with fold B (Fig. 4C). However, we cannot exclude the possibility that this reconstruction is an artefact arising from suboptimal classification of filament segments. Folds A and B are almost identical at residues G36‐V55, and V63‐A78, with some flexibility in the β‐turn at residues E57‐E61. However, comparing the more compact fold B to fold A, a flip in K80 from the hydrophobic core towards the solvent results in a sharp turn at T81 and a shift by three residues in the packing of β4 against β7 (Fig. 3, Fig. S7).

### Cryo‐EM structure of type 3 α‐synuclein filaments

**Fig. 5:**
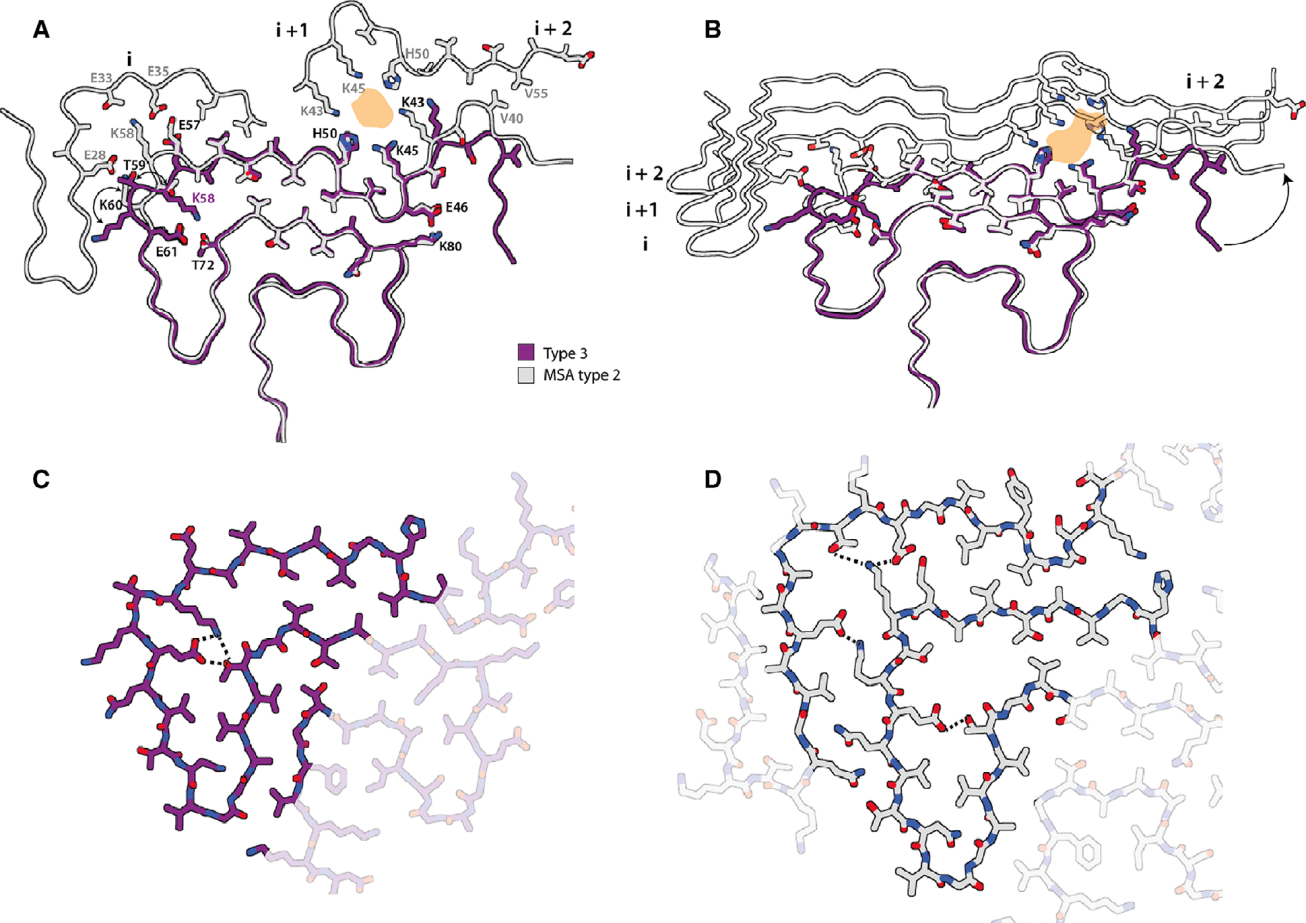
Comparison of type 3 filament with protofilament IIB from MSA case 5. (A) Atomic model of the type 3 filament (purple) overlaid with the model of protofilament IIB_2_ from MSA case 5. The additional density at the protofilament interface of MSA type II filaments is shown in orange. (B) Cartoon view of one rung of type 3 filaments overlaid with one rung of protofilament IIB and three rungs of protofilament IIA of MSA case 5. Residues on MSA protofilament IIA that interact with the rung of protofilament IIB shown are highlighted with sticks. (C) Close up all‐atom view of the hydrogen‐bonding network (yellow dashed) between K58, E61 and T72 in type 3 filaments. (D) As in (C), but for protofilaments IIA and IIB in MSA filaments.

Type 3 filaments consist of a single protofilament that extends from G36‐Q99 and comprises 10 β‐sheets (β1‐10) (Fig. 4). Residues 46–99 form a Greek key motif, as described before [29], with a salt bridge between E46 and K80. This motif is preceded by a β‐arch formed by residues Y39‐T44 and Y39‐E46. The density between residues 36 and 39 is more smeared. Two stretches of elongated, smeared densities, possibly originating from parts of the N‐terminus of α‐synuclein, are observed in front of β1 in the β‐arch and β4 in the Greek key motif. An additional fuzzy density is observed in front of the side chains of K43, K45 and H50. Whereas filament types 1 and 2 did not resemble the four protofilaments observed in MSA, type 3 filaments were almost identical to protofilament IIB_2_, with an r.m.s.d. between atomic coordinates of 1.02 Å (Fig. 5). However, in MSA filaments, K58 is flipped away from the core of the protofilament to form a salt bridge with T33 of the opposing protofilament, whereas K58 forms part of the protofilament core in type 3 filaments. Minor rearrangements occur near V40, which is also involved in inter‐protofilament packing in MSA filaments. Interestingly, the position of the density of the unidentified co‐factor at the inter‐protofilament interface of type II filaments coincides with the fuzzy density in front of K43, K45 and H50. Type 3 filaments are almost identical to the narrow protofilament formed upon *in vitro* assembly of recombinant H50Q α‐synuclein [37], with an r.m.s.d. between atomic coordinates of 0.62 Å (Fig. 5; Fig. S10).

### Cryo‐EM structures of α‐synuclein filaments from second‐generation seeded aggregation

To further explore the effects of buffer conditions on seeded aggregation, we incubated seeds from MSA case 5 with recombinant human α‐synuclein in phosphate‐buffered saline (PBS). We previously observed that the density for the additional molecules at the interface between protofilaments in our reconstructions of MSA filaments [32] overlaps with similar densities in reconstructions of *in vitro* aggregated recombinant α‐synuclein, which have been attributed to phosphate ions [25, 30]. Since the additional density in MSA filaments could accommodate two phosphate ions, we supplemented PBS with 1 mm pyrophosphate. However, by negative‐stain imaging, the seeded assemblies were indistinguishable from those formed using PBS without pyrophosphate. We then performed second‐generation seeded assembly, in which the aggregates from the assembly in PBS‐pyrophosphate were used as seed. Cryo‐EM structure determination of the seeded assemblies confirmed the faithful propagation of type 3 filaments, with a larger proportion of type 3 doublet filaments (~ 5%) (Fig. 4; Fig. S8).

## Discussion

**Fig. 6:**
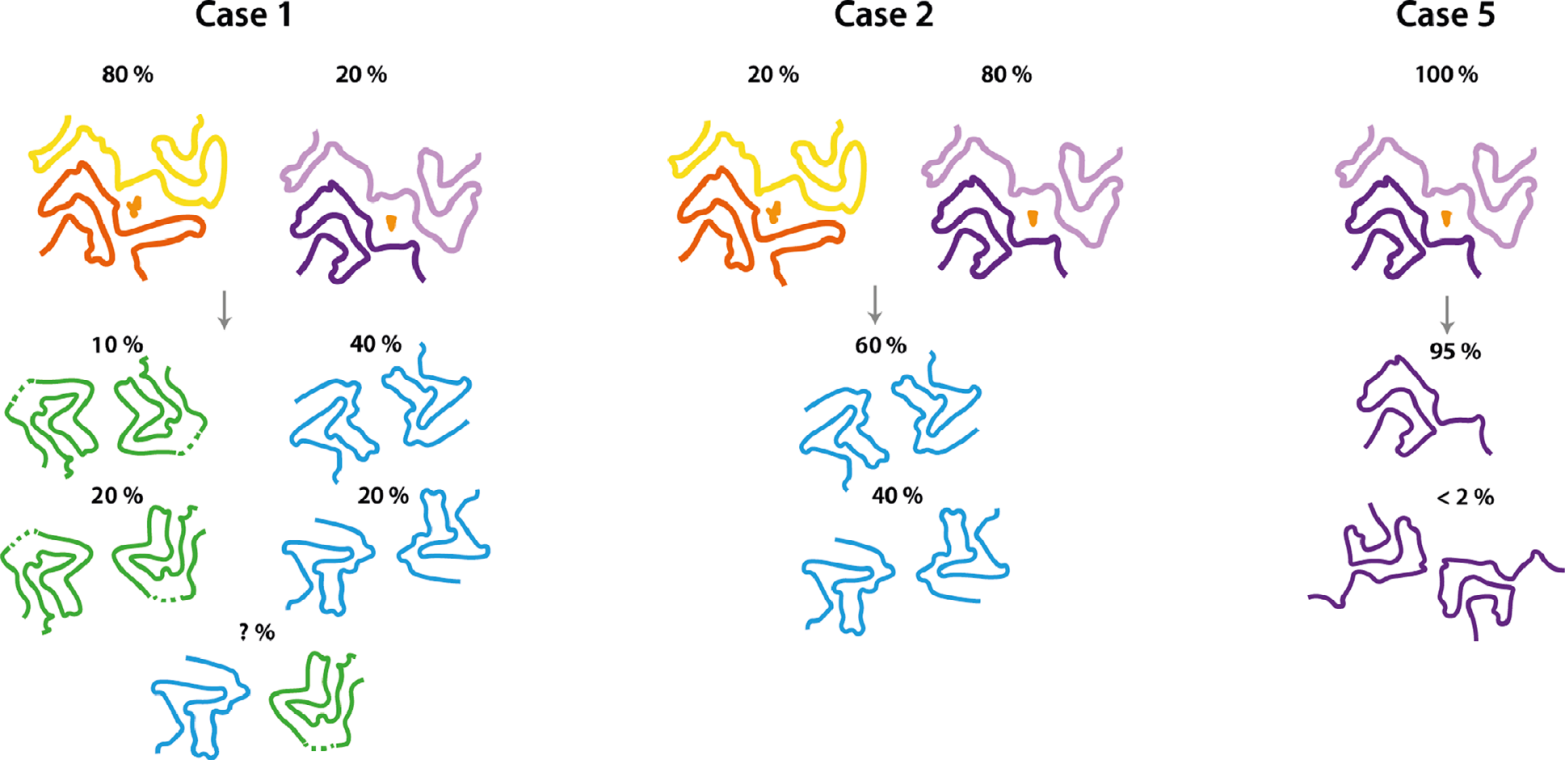
Summary of MSA seeded aggregation experiments. Cartoon illustrations show the structures of MSA type I and type II filaments and their relative quantities in MSA cases 1, 2 and 5 at the top, and the products of seeded aggregation underneath.

We show here that the structures of seeded assemblies of wild‐type recombinant human α‐synuclein differ from those of seeds that were extracted from the brains of individuals with MSA (Fig. 6). We used the assembly conditions of Shahnawaz *et al*. [23] who reported that PMCA, using cerebrospinal fluid as seed and recombinant α‐synuclein as substrate, can discriminate between PD and MSA. It remains to be seen if α‐synuclein seeds from PD brain yield structures that are different from those described here. Nevertheless, our results raise important questions for the study of amyloid structures and prion processes.

Amyloid filaments are structurally versatile, with the same amino acid sequences being able to adopt different structures [33, 38]. Moreover, the cryo‐EM structures of tau, β‐amyloid and α‐synuclein filaments from human brain are different from those of recombinant proteins assembled *in vitro* [33, 39, 40]. The present findings demonstrate that, even when using brain‐derived filament preparations to seed *in vitro* assembly, the resulting structures do not necessarily replicate those of the seeds.

When using seeds from MSA cases 1 and 2, which contain a mixture of type I and type II filaments, and recombinant human α‐synuclein as substrate, we observed the formation of type 1 and type 2 filaments. When using seeds from MSA case 5, with only type II filaments, we observed the formation of filaments of type 3. These observations suggest that in seeded assemblies, type I filaments overshadow type II MSA filaments, despite the observation that seeds of case 5 resulted in a faster and stronger increase in thioflavin‐T fluorescence compared to seeds from cases 1 and 2. The possibility that different conformational strains have different seeding potencies has implications for the interpretation of prion propagation assays.

It is commonly assumed that self‐propagation of strains occurs through templated incorporation of monomers at the ends of amyloid filaments. Indeed, following sonication, α‐synuclein filaments had increased seeding potencies [41, 42]. However, it is unclear how this could explain the formation of type 1 and type 2 filaments with markedly different protofilament folds, when compared to MSA filaments. Each prion strain is believed to comprise a large number of conformationally distinct assemblies (also known as clouds), often with a dominant conformer that propagates under host selection [43, 44]. Our work on tau and α‐synuclein assemblies has shown the presence of only one or two major filament types in the brains from patients at end‐stage disease [32, 33]. It is possible that type 1 and type 2 filaments were present in the filament preparations from MSA brains, but not numerous enough to be detected by cryo‐EM [32]. We previously demonstrated that tau structures that only made up around 3% of filaments can be detected [45], indicating that, if present in MSA brains, type 1 and type 2 α‐synuclein filaments are infrequent.

Type 3 filaments, which assembled from MSA type II seeds, fit the model of structural equivalence between seeds and seeded assemblies better than type 1 and type 2 filaments, because their structure overlaps almost completely with that of type IIB protofilaments from the putamen of patients with MSA. We previously attributed additional cryo‐EM densities at the inter‐protofilament interfaces of type I and type II MSA filaments to negatively charged, nonproteinaceous molecules. It is possible that the absence of these molecules in the seeded assembly experiments led to the formation of a structure that represents only half of the seed structures. These findings indicate that protofilament IIB, but not IIA, can form from recombinant α‐synuclein through seeded assembly without added cofactor.

Abundant GCIs in oligodendrocytes are the major neuropathological hallmark of MSA [46]. Thus, differences in the cellular milieu between oligodendrocytes and other brain cells may play a role in the seeded aggregation of MSA filaments. Oligodendrocytes have been shown to transform misfolded α‐synuclein into a GCI‐like strain [47].

Besides the possible incorporation of other molecules in α‐synuclein filaments from human brain, it is also conceivable that recombinant α‐synuclein is not able to form MSA filaments. Truncation and post‐translational modifications of α‐synuclein may be needed [9, 10]. In α‐synuclein filament preparations from the putamen of patients with MSA, mass spectrometry identified N‐terminal acetylation, C‐terminal truncation, ubiquitination at K6 K12, K21, acetylation at K21 K23 K32 K34 K45 K58 K60 K80 and K96 and phosphorylation at Y39, T59, T64, T72 and T81 [32]. It is not known if these modifications occur prior to, during or after filament assembly, and if or how they may affect filament conformations. Assembly of recombinant wild‐type human α‐synuclein using seeds of α‐synuclein phosphorylated at Y39 gave rise to filaments with a different fold from that of the seeds [48]. Moreover, C‐terminal truncation of recombinant α‐synuclein has been shown to promote filament assembly *in vitro* [49]; inhibiting C‐terminal truncation in transgenic mouse models of MSA has been reported to reduce pathology [10, 50]. It has also been shown that interactions with lipids, DNA, RNA, iron and phosphate promote α‐synuclein aggregation *in vitro*, and similar interactions could be important for the formation of MSA filaments in brain [51, 52, 53].

Identification of the factors that govern the replication of conformational prion strains will be essential for our understanding of propagation of the distinct proteinopathies. Meanwhile, the relevance of the structures of amyloids assembled from recombinant protein seeds and the results of self‐propagation studies should be interpreted with care.

## Materials and methods

### Expression and purification

α‐Synuclein was expressed and purified, essentially as described [54]. Briefly, plasmid pRK172 encoding a cDNA for full‐length, wild‐type human α‐synuclein was transformed into *E*. *coli* BL21(DE3)‐gold (Agilent Technologies LDA UK Limited, Stockport, UK). Cells were cultured in 2xTY, 5 mm MgCl_2_ and 100 mg·L^−1^ ampicillin at 37 °C until an OD_600_ of 0.7 was reached; α‐synuclein expression was then induced with 1 mm IPTG. After 4 h, cells were harvested by centrifugation and resuspended in buffer A [50 mm Tris/HCl, pH 7.5, 10 mm EDTA, 2.5 mm TCEP (Sigma‐Aldrich, Gillingham, UK), 0.1 mm AEBSF (Sigma‐Aldrich), 40 μg·mL^−1^ DNase and 10 μg·mL^−1^ RNase (Sigma‐Aldrich), supplemented with cOmplete EDTA‐free Protease Inhibitor Cocktail (Roche, Welwyn Garden City, UK)]. They were lysed by sonication on ice using a Sonics VCX‐750 Vibra Cell Ultra Sonic Processor for 5 min (5 s on, 10 s off) at 40% amplitude. The lysates were centrifuged at 17 000 ***g*** for 40 min at 4 °C, filtered with a 0.45 μm cut‐off filter, loaded onto an anion exchange Sepharose 26/10 Q column (GE Healthcare, Chalfont Saint Giles, UK) and eluted with a 0–1 m NaCl gradient. Fractions containing α‐synuclein were precipitated using ammonium sulphate (0.3 g·mL^−1^) for 30 min at 4 °C and centrifuged at 16 000 ***g*** for 30 min at 4 °C. The resulting pellets were resuspended in buffer B (PBS, 0.1 mm AEBSF, supplemented with cOmplete EDTA‐free Protease Inhibitor Cocktail), loaded onto a HiLoad 16/60 Superdex (GE Healthcare) column equilibrated in buffer B and eluted using a flow rate of 1 mL·min^−1^. The purity of α‐synuclein was analysed by SDS/PAGE and protein concentrations determined spectrophotometrically using an extinction coefficient of 5600 M^−1^·cm^−1^.

### Extraction of MSA filament seeds

The filament preparations used in this study have been described [32]. Briefly, frozen putamen from MSA cases 1, 2 and 5 was homogenized in 20% vol (w/v) extraction buffer (10 mm Tris/HCl, pH 7.5, 0.8 m NaCl, 1 mm EGTA, 10% sucrose, 2% sarkosyl, pH 7.5) and incubated for 30 min at 37 °C. The homogenates were centrifuged for 10 min at 10 000 ***g*** at room temperature, followed by a 20 min spin of the resulting supernatants at 100 000 ***g***. The pellets were resuspended in 500 μL·g^−1^ extraction buffer and centrifuged at 3000 ***g*** for 5 min to remove large contaminants. The supernatants were diluted in 50 mm Tris/HCl, pH 7.5, containing 150 mm NaCl, 10% sucrose and 0.2% sarkosyl, and centrifuged at 166 000 ***g*** for 30 min. Sarkosyl‐insoluble pellets were resuspended in 50 μL·g^−1^ tissue and filament concentrations estimated by negative‐stain EM. Prior to seeded assembly experiments, pellets were centrifuged at 2000 ***g*** for 5 min, the resulting supernatants were diluted 10‐fold, and sonicated in an Eppendorf tube using a VialTweeter (Hielscher Ultrasonics GmbH, Teltow, Germany) at a cumulative power of 100 W. Sonication did not alter the structure of the seeds, as suggested by negative‐stain EM (Fig. 1; Fig. S1), and as confirmed by cryo‐EM 2D class averages of the seeds before and after sonication (Fig. 1; Fig. S1).

### Seeded assembly

Purified recombinant α‐synuclein was centrifuged at 20 000 ***g*** for 1 h to remove potential aggregates. 70 μm recombinant α‐synuclein was incubated with 2 μm MSA seeds (as assessed by negative‐stain EM) in 100 mm PIPES pH 6.5, 500 mm NaCl, 0.05% NaN_3_ and 5 μm thioflavin‐T, in a final volume of 200 µL per experiment. Controls used buffer without seeds. Seeded assembly proceeded for 120 h at 37 °C in a FLUOstar Omega (BMG Labtech, Aylesbury, UK) microplate reader where the samples were alternatingly shaken for 1 min at 400 rpm, and left to rest for 1 min, during which fluorescence was measured.

For cryo‐EM, seeded assembly conditions were identical, but no thioflavin‐T was added to the buffer and the samples were shaken continuously for 72 h. Seeded assembly experiments for cryo‐EM were also performed in PBS buffer, supplemented with 1 mm pyrophosphate and 0.05% NaN_3_. The resulting filaments were pelleted, resuspended in 200 μL and sonicated as described above, and then used as seeds (2 µm) for a second‐generation seeded assembly experiment with recombinant α‐synuclein (70 µm) in the same PBS buffer.

### Cryo‐EM grid preparation and imaging

Prior to freeze plunging, filaments were pelleted for 45 min at 100 000 ***g*** and resuspended at 100 μm α‐synuclein in 50 mm Tris, pH 7.5, 50 mm NaCl. Four microlitres of sample was applied to glow‐discharged 1.2/1.3 holey carbon‐coated gold grids (Quantifoil AU R1.2/1.3, 300 mesh) for 30 s, blotted with filter paper for 3.5 s and plunge‐frozen in liquid ethane using an FEI Vitrobot Mark IV. Filaments were imaged on a Thermo Fischer Titan Krios microscope operating at 300 kV equipped with a Gatan K2 Summit direct detector in counting mode and a GIF Quantum energy filter (Gatan) with a slit width of 20 eV to remove inelastically scattered electrons. Acquisition details are given in Table 1.

**Table 1:** Cryo‐EM data collection, refinement and validation statistics.

Data collection and processing	Type 1A Case 2 (EMD‐12264) (PDB 7NCA)	Type 2A Case2 (EMD‐12265) (PDB 7NCG)	Type 1B Case 1 (EMD‐12266) (PDB 7NCH)	Type 2B Case 1 (EMD‐12267) (PDB 7NCI)	Type 2A/B Case 1 (EMD‐12268) (PDB 7NCJ)	Type 3 Case 5 (EMD‐12269) (PDB 7NCK)	Type 3 Case 5 Second generation	Type 3 doublet Case 5 Second generation
Magnification	105 000×	105 000×	105 000×	105 000×	105 000×	105 000×	105 000×	105 000×
Voltage (kV)	300	300	300	300	300	300	300	300
Detector	K2	K2	K2	K2	K2	K2	K2	K2
Electron exposure (e–/Å^2^)	32.6	32.6	36.7	36.7	36.7	37.5	37.0	37.0
Defocus range (μm)	−1.5 to −2.8	−1.5 to −2.8	−1.5 to −2.8	−1.5 to −2.8	−1.5 to −2.8	−1.5 to −2.8	−1.5 to −2.8	−1.5 to −2.8
Pixel size (Å)	1.14	1.14	1.1	1.1	1.1	1.14	1.14	1.14
Micrographs	1294	1294	2172	2172	2172	1265	1317	1317
Symmetry imposed	C2	C2	C2	C2	C1	C1	C1	C2
Initial particle images (no.)	287 364	287 364	441 592	441 592	441 592	122 831	270 003	270 003
Final particle images (no.)	67 619	82 474	33 479	87 092	57 358	69 490	18 691	82 474
Map resolution (FSC = 0.143) (Å)	3.47	3.43	3.84	3.55	4.23	3.18	3.54	4.40
Map resolution range (Å)	2.8–11	3.2–6.3	3.5–10	3.3–18	4.0–14	2.7–5.5	NA	NA
Helical twist (º)	−1.04	−0.95	−0.86	−0.77	−0.86	−0.95	−0.95	−1.52
Helical rise (Å)	4.75	4.75	4.78	4.75	4.80	4.75	4.75	4.75
Refinement								
Initial model used (PDB code)	6UFR	6UFR	6SSX	6SST	6SST/6UFR	6PEO		
Model resolution (FSC = 0.5) (Å)	3.4	3.7	5.4	4.6	5.4	3.5		
Map sharpening *B* factor (Å^2^)	−79.5	−68.3	−105.7	−81.9	−107.8	−56.6		
Model composition								
Nonhydrogen atoms	5052	5032	5496	5496	5274	2652		
Protein residues	732	732	816	816	774	384		
Ligands	0	0	0	0	0	0		
R.m.s. deviations								
Bond lengths (Å)	0.011	0.012	0.013	0.011	0.009	0.010		
Bond angles (°)	1.966	2.133	1.606	2.118	1.432	2.002		
Validation								
MolProbity score	0.88	1.12	1.03	1.12	1.06	0.97		
Clashscore	0.00	0.38	0.00	0.27	0.18	0.18		
Poor rotamers (%)	0.19	0.00	0.19	0.00	0.76	0.37		
Ramachandran plot								
Favoured (%)	94.49	92.23	90.62	91.15	92.01	94.09		
Allowed (%)	5.51	7.77	9.38	8.85	7.72	5.91		
Disallowed (%)	0.00	0.00	0.00	0.00	0.27	0.00		

### Helical reconstruction

Filaments were reconstructed in RELION‐3.1 [55] using helical reconstruction [35]. Movie frames were corrected for beam‐induced motions and dose‐weighted in RELION using its own motion‐correction implementation [56]. Non‐dose‐weighted micrographs were used for CTF estimation with CTFFIND‐4.1 [57]. Filaments were picked manually, ignoring those without a clear twist. Initially, particle segments were extracted using a box size of 550 pixels and an interbox distance of 14 Å and downscaled to 225 pixels for 2D classification. For filaments formed from the seeds of MSA cases 1 and 2, filament types 1 and 2 were separated at this initial 2D classification stage. Cross‐over distances were obtained by manual measurements in the micrographs and used to calculate initial estimates for the helical twist of the different filament types: −1.0º for type 1; −0.8º for type 2; and −1.5° for type 3, assuming a helical rise of 4.75 Å. *De novo* 3D initial models were then constructed from 2D class averages representing one whole cross‐over of the different filament types using the relion_helix_inimodel2d program [58]. Subsequently, segments were re‐extracted without down‐sampling in boxes of 256 × 256 pixels for use in 3D auto‐refinements and classifications. Several rounds of refinements were performed, while progressively increasing the resolution of the starting model from 10 Å to 4.5 Å and switching on optimization of the helical rise and helical twist once β‐strands were separated in the starting model. For filaments from seeds of MSA case 1, additional 3D classifications focussed classifications on exterior regions of the filament were used to distinguish the presence of minority polymorphs (with protofilament fold B as described in the main text). Final reconstructions were obtained after Bayesian polishing and CTF refinement, followed by 3D auto‐refinement, a 3D classification step without alignment to select the segments contributing to the best classes, a final round of 3D auto‐refinement and standard RELION postprocessing with a soft solvent mask that extended to 20 % of the box height.

### Atomic modelling

Atomic models of the filaments were built *de novo* in *Coot* [59] using the maps of the data set for MSA case 2 for type 1 and type 2 filaments with protofilament fold A, and maps of the data set for MSA case 2 for type 1 and type 2 filaments with protofilament fold B. For protofilament fold A, the atomic model with PDB‐ID 6UFR of E46K α‐synuclein [36] was used as guide. For type 3 filaments, the atomic model with PDB‐ID 6PEO [37] of H50Q α‐synuclein was used. Models comprising 6 β‐sheet rungs were refined in real‐space using ISOLDE [60], with interactive flexible molecular dynamics to obtain optimal β‐sheet packing chemistry. The resulting models were validated with MolProbity [61]. Details about the atomic models are described in Table 1.

The schematics in Figs S7e‐f and S10 were made with T. Nakane's atoms2svg.py script, which is publicly available from https://doi.org/10.5281/zenodo.4090924.

## Conflict of interest

The authors declare no conflict of interest.

## Author contributions

SL performed seeded aggregation and cryo‐EM experiments and analysed the data, with contributions from MS, MG and SHWS; TM, SM, TT, TA, KH, MY, AT and MH identified patients, performed neuropathology and extracted α‐synuclein filaments from MSA cases; SHWS and MG supervised the project; SL, MG and SHWS wrote the manuscript, with inputs from all authors.

## Ethical review processes and informed consent

The procedures for the extraction of MSA filaments from human brain were approved through the ethical review process at Tokyo Metropolitan Institute of Medical Science and the study methodologies conformed the to the standards set by the declaration of Helsinki. Informed written consent was obtained from the patients’ next of kin.

## Supporting information


**Fig. S1.** Purified filaments from MSA case 5. (a) Sarkosyl insoluble pellet before sonication; and (b) after sonication (scale bar = 200 nm). Cryo‐EM 2D class averages of MSA case 5 purified filaments before sonication (c) and after sonication (d).
**Fig. S2.** Additional cryo‐EM data on type 1 and type 2 filaments with protofilament fold A. (a) Electron micrograph of seeded assemblies using filament preparations from MSA case 2 as the seed. Type 1 and type 2 filaments are indicated with white arrows. Scale bar, 50 nm. (b) 2D class averages of type 1 (left) and type 2 (right) filaments with two protofilaments of fold A in a box spanning 280 A. (c) Local resolution maps for type 1 (top) and type 2 (bottom) filaments, with the legend indicating resolutions in A. (d) Side view of the 3D reconstructions for type 1 (left) and type 2 (right) filaments, showing clear separation of β‐strands along the helical axis. (e) FSC curves for type 1 filaments (left) and type 2 filaments (right) with two protofilaments of fold A between two independently refined half‐maps (black), of the final cryo‐EM reconstruction and refined atomic model (red), of the first half map and the atomic model refined against the first half map (blue), and of the atomic model that was refined against the first half‐map against the second half‐map (yellow dashed).
**Fig. S3.** Cryo‐EM structures of type 1 and type 2 filaments with protofilament fold A assembled using seeds from MSA case 1. (a) Central slice of the 3D map for type 1 filaments. (b) Side view of the 3D reconstruction of type 1 filaments. (c–d) As (a–b), but for type 2 filaments.
**Fig. S4.** Comparison of protofilament fold A with PDB‐entry 6UFR of assembled recombinant E46K α‐synuclein. (a) Atomic model of protofilament fold A (blue) overlaid with one protofilament from PDBentry 6UFR (grey). (b) Comparison of the interface between two protofilaments with fold A in type 1 filaments and those from PDB entry 6UFR, with the same colour scheme as in (a). (c) Zoomed‐in view of the interface, with salt bridges between K45 and E47 in PDB‐entry 6UFR and between E46 and K58 in type 1 filaments highlighted in grey and blue, respectively.
**Fig. S5.** Additional cryo‐EM data on type 1 and type 2 filaments with protofilament fold B. (a) 2D class averages of type 1 filaments with two protofilaments of fold B (b) Local resolution map for type 1 filaments with two protofilaments of fold B with the colour map indicating resolutions in A. (c) Side view of the 3D reconstructions of type 1 filaments with two protofilaments of fold B. (d‐f) as (a‐c) but for type 2 filaments with two protofilaments of fold B. (g‐i) as (a‐c) but for type 2 filaments with one protofilament of fold A and one protofilament of fold B. (j‐l) Fourier shell correlation curves for type 1 filaments with two protofilaments of fold B (j), type 2 filaments with two protofilaments of fold B (k) and type 2 filaments with one protofilament of fold A and one protofilament of fold B (l). Fourier shell correlation curves are shown between two independently refined half‐maps (black) of the final cryo‐EM reconstruction and refined atomic model (red), of the first half map and the atomic model refined against the first half map (blue), and of the atomic model that was refined against the first half‐map against the second half‐map (yellow dashed).
**Fig. S6.** Comparison of protofilament A with PDB‐entry 6SSX of recombinant wild‐type α‐synuclein. (a) Atomic model of protofilament fold A (blue) overlaid with one protofilament from PDBentry 6SSX (grey). (b) Comparison of the interface between two protofilaments with fold A in type 1 filaments and those from PDB entry 6UFR, with the same colour scheme as in (a). (c) Zoomed‐in view of the interface, with salt bridges between K45 and E47 in PDB‐entry 6UPR and between E46 and K58 in type 1 filaments highlighted in grey and blue, respectively.
**Fig. S7.** Comparison of protofilament folds A and B. (a) Atomic model of protofilament fold A (blue) overlaid with protofilament fold B (green) (b,c) As in (a), but showing all‐atom representation for different residues. (d,e) Schematic representations of protofilament folds A and B. Each amino acid residue is represented with its one‐letter code in a circle. Positively charged amino acids are shown in blue, negatively charged ones in red, polar ones in green, hydrophobic ones in white, and glycines in pink.
**Fig. S8.** Additional cryo‐EM data on type 3 filaments. (a) Electron micrograph of case 5. The scale bar indicates 50 nm. (b) 2D class averages of type 3 filaments in a box spanning 280 A. (c) Local resolution map, with the colour map indicating resolutions in A. (d) Fourier shell correlation curves between two independently refined halfmaps (black), of the final cryo‐EM reconstruction and the refined atomic model (red), of the first half map and the atomic model refined against the first half map (blue), and of the atomic model that was refined against the first half‐map against the second half‐map (yellow dashed). (e) Side view of the 3D reconstruction, showing separation of β‐strands along the helical axis.
**Fig. S9.** Second‐generation type 3 filaments. (a) Central slice of the 3D map of the type 3 filaments from the second generation of seeding. (b) Side view of the 3D reconstruction of the same type 3 filaments. (c‐d) As in (a‐b), but for the doublets of type 3 filaments.
**Fig. S10.** Comparison of type 3 filament with PDB entry 6PEO of assembled recombinant H50Q α‐synuclein. (a), All‐atom view of the type 3 filament (purple) aligned with PDB‐entry 6PEO (grey). (b), Schematic representation of the type 3 filament.

## Data Availability

Cryo‐EM maps and raw micrographs have been deposited in the Electron Microscopy Data bank (EMDB) under the accession numbers EMD‐12264, EMD‐12265, EMD‐12266, EMD‐12267, EMD‐12268, EMD‐12269 for types 1A, 2A, 1B, 2B, 2A/B and 3, respectively. The corresponding atomic models have been deposited in the Protein Data Bank (PDB) under the accession numbers: 7NCA, 7NCG, 7NCH, 7NCI, 7NCJ and 7NCK for types 1A, 2A, 1B, 2B, 2A/B and 3, respectively. Additional data will be available from the corresponding authors upon reasonable request.
