# Supplementary material for: Seeded assembly in vitro does not replicate the structures of α‐synuclein filaments from multiple system atrophy
Source: FEBS Open Bio. 2021 Feb 24;11(4):999–1013. doi: 10.1002/2211-5463.13110 (PMC8016116; doi:10.1002/2211-5463.13110)
Supplement: Supplementary file 1 — Fig. S1. Purified filaments from MSA case 5. (a) Sarkosyl insoluble pellet before sonication; and (b) after sonication (scale bar = 200 nm). Cryo‐EM 2D class averages of MSA case 5 purified filaments before sonication (c) and after sonication (d). Fig. S2. Additional cryo‐EM data on type 1 and type 2 filaments with protofilament fold A. (a) Electron micrograph of seeded assemblies using filament preparations from MSA case 2 as the seed. Type 1 and type 2 filaments are indicated with white arrows. Scale bar, 50 nm. (b) 2D class averages of type 1 (left) and type 2 (right) filaments with two protofilaments of fold A in a box spanning 280 A. (c) Local resolution maps for type 1 (top) and type 2 (bottom) filaments, with the legend indicating resolutions in A. (d) Side view of the 3D reconstructions for type 1 (left) and type 2 (right) filaments, showing clear separation of β‐strands along the helical axis. (e) FSC curves for type 1 filaments (left) and type 2 filaments (right) with two protofilaments of fold A between two independently refined half‐maps (black), of the final cryo‐EM reconstruction and refined atomic model (red), of the first half map and the atomic model refined against the first half map (blue), and of the atomic model that was refined against the first half‐map against the second half‐map (yellow dashed). Fig. S3. Cryo‐EM structures of type 1 and type 2 filaments with protofilament fold A assembled using seeds from MSA case 1. (a) Central slice of the 3D map for type 1 filaments. (b) Side view of the 3D reconstruction of type 1 filaments. (c–d) As (a–b), but for type 2 filaments. Fig. S4. Comparison of protofilament fold A with PDB‐entry 6UFR of assembled recombinant E46K α‐synuclein. (a) Atomic model of protofilament fold A (blue) overlaid with one protofilament from PDBentry 6UFR (grey). (b) Comparison of the interface between two protofilaments with fold A in type 1 filaments and those from PDB entry 6UFR, with the same colour scheme as in (a). (c) [file FEB4-11-999-s001.pdf]

Supplementary Figures

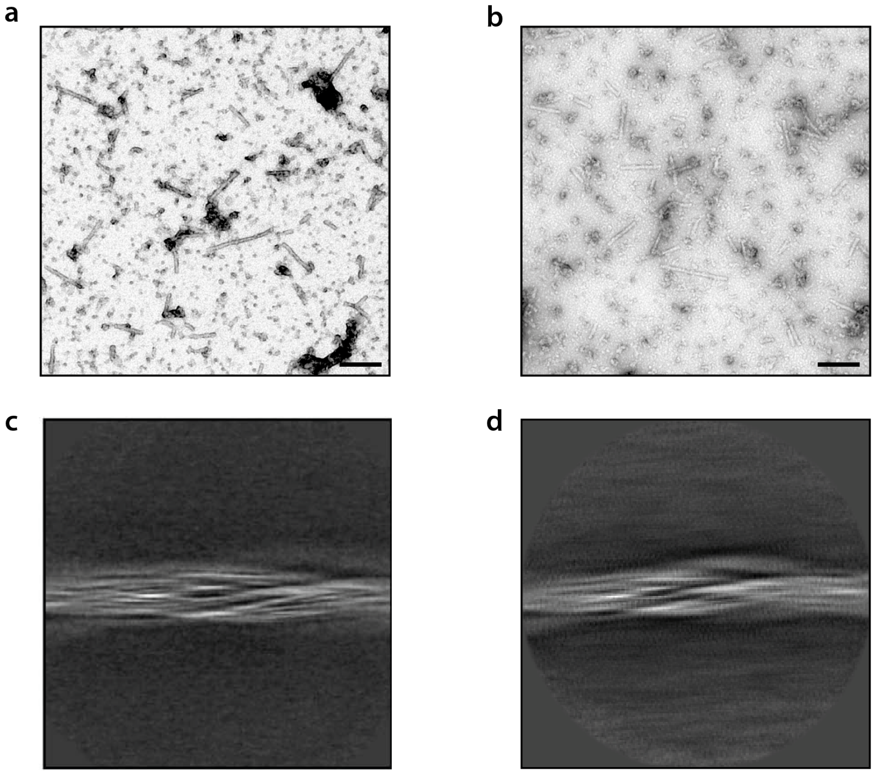

Supplementary Figure 1. Purified filaments from MSA case 5

(a) Sarkosyl insoluble pellet before sonication; and **(b)** after sonication (scale bar = 200 nm). Cryo-EM 2D class averages of MSA case 5 purified filaments before sonication **(c)** and after sonication **(d)**.

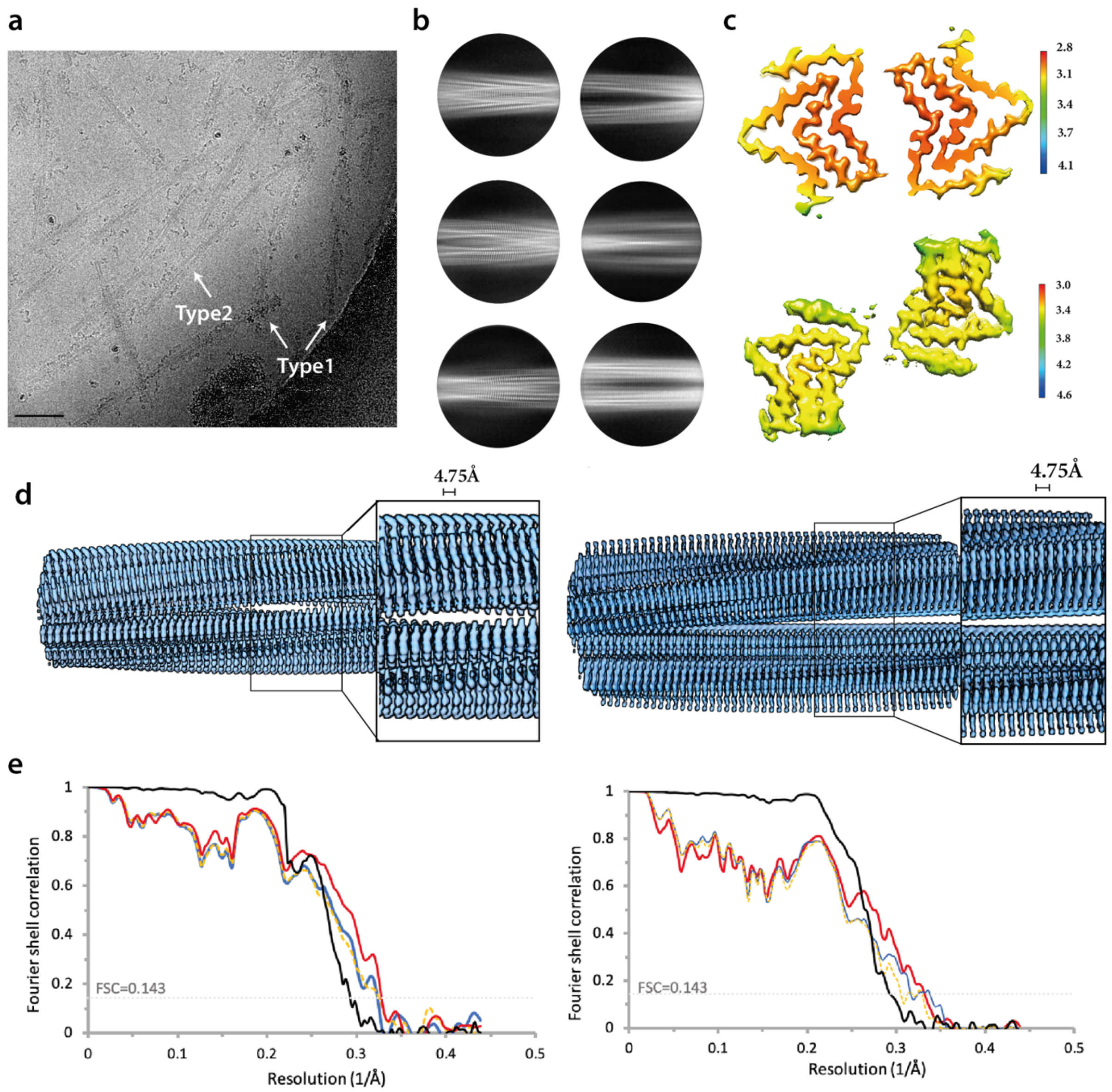

Supplementary Figure 2. Additional cryo-EM data on type 1 and type 2 filaments with protofilament fold A.

(a) Electron micrograph of seeded assemblies using filament preparations from MSA case 2 as the seed. Type 1 and type 2 filaments are indicated with white arrows. Scale bar, 50 nm. **(b)** 2D class averages of type 1 (left) and type 2 (right) filaments with two protofilaments of fold A in a box spanning 280 Å. **(c)** Local resolution maps for type 1 (top) and type 2 (bottom) filaments, with the legend indicating resolutions in Å. **(d)** Side view of the 3D reconstructions for type 1 (left) and type 2 (right) filaments, showing clear separation of β-strands along the helical axis. **(e)** FSC curves for type 1 filaments (left) and type 2 filaments (right) with two protofilaments of fold A between two independently refined half-maps (black), of the final cryo-EM reconstruction and refined atomic model (red), of the first half map and the atomic

model refined against the first half map (blue), and of the atomic model that was refined against the first half-map against the second half-map (yellow dashed).

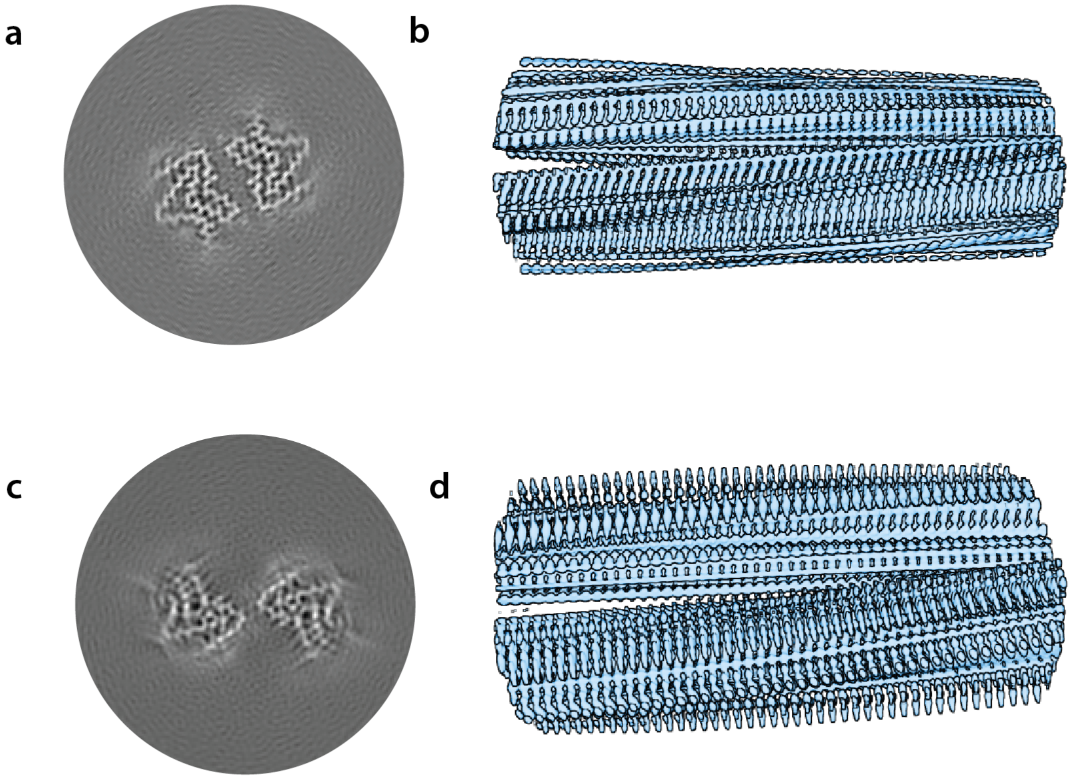

Supplementary Figure 3. Cryo-EM structures of type 1 and type 2 filaments with protofilament fold A assembled using seeds from MSA case 1.

(a) Central slice of the 3D map for type 1 filaments. **(b)** Side view of the 3D reconstruction of type 1 filaments. **(c-d)** As (a-b), but for type 2 filaments.

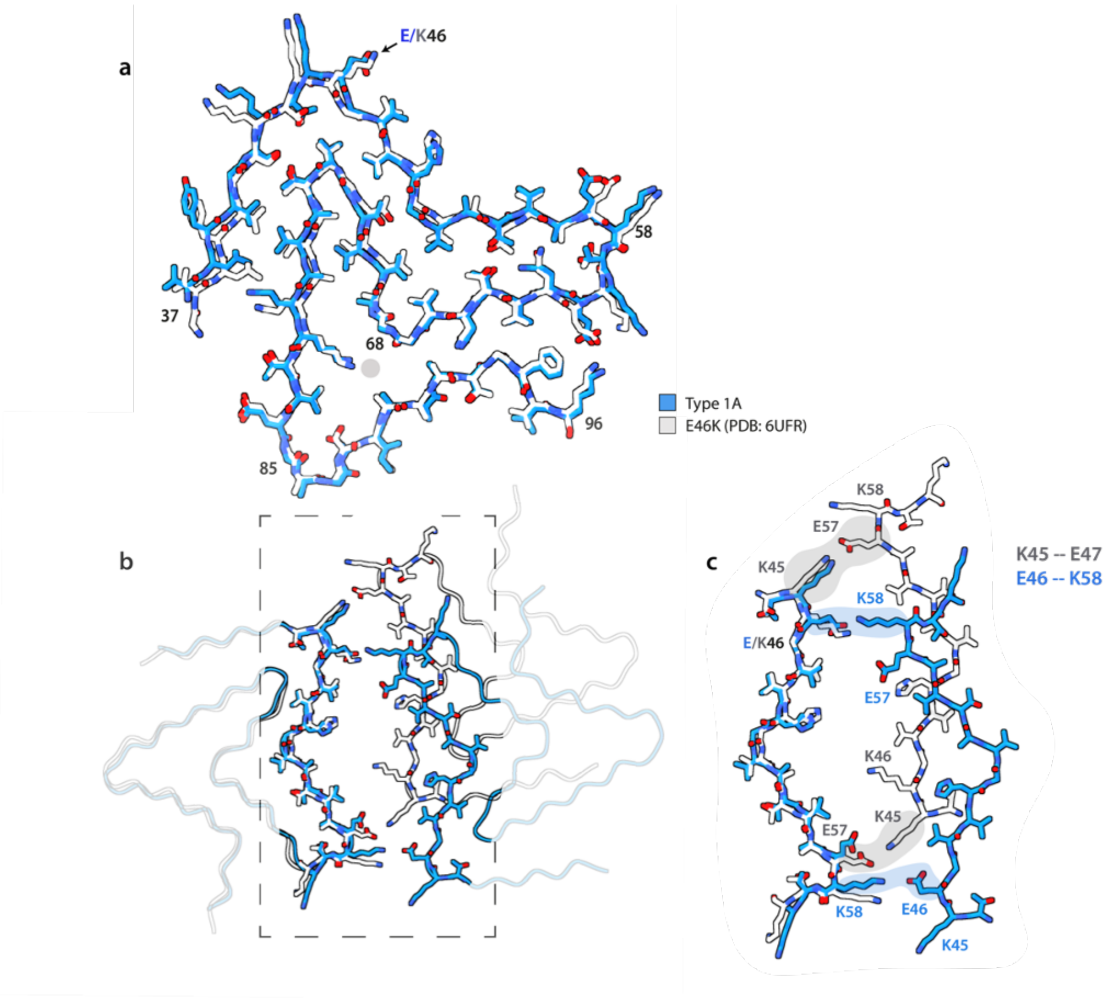

Supplementary Figure 4. Comparison of protofilament fold A with PDB-entry 6UFR of assembled recombinant E46K α-synuclein.

(a) Atomic model of protofilament fold A (blue) overlaid with one protofilament from PDB-entry 6UFR (grey). **(b)** Comparison of the interface between two protofilaments with fold A in type 1 filaments and those from PDB entry 6UFR, with the same colour scheme as in (a). **(c)** Zoomed-in view of the interface, with salt bridges between K45 and E47 in PDB-entry 6UFR and between E46 and K58 in type 1 filaments highlighted in grey and blue, respectively.

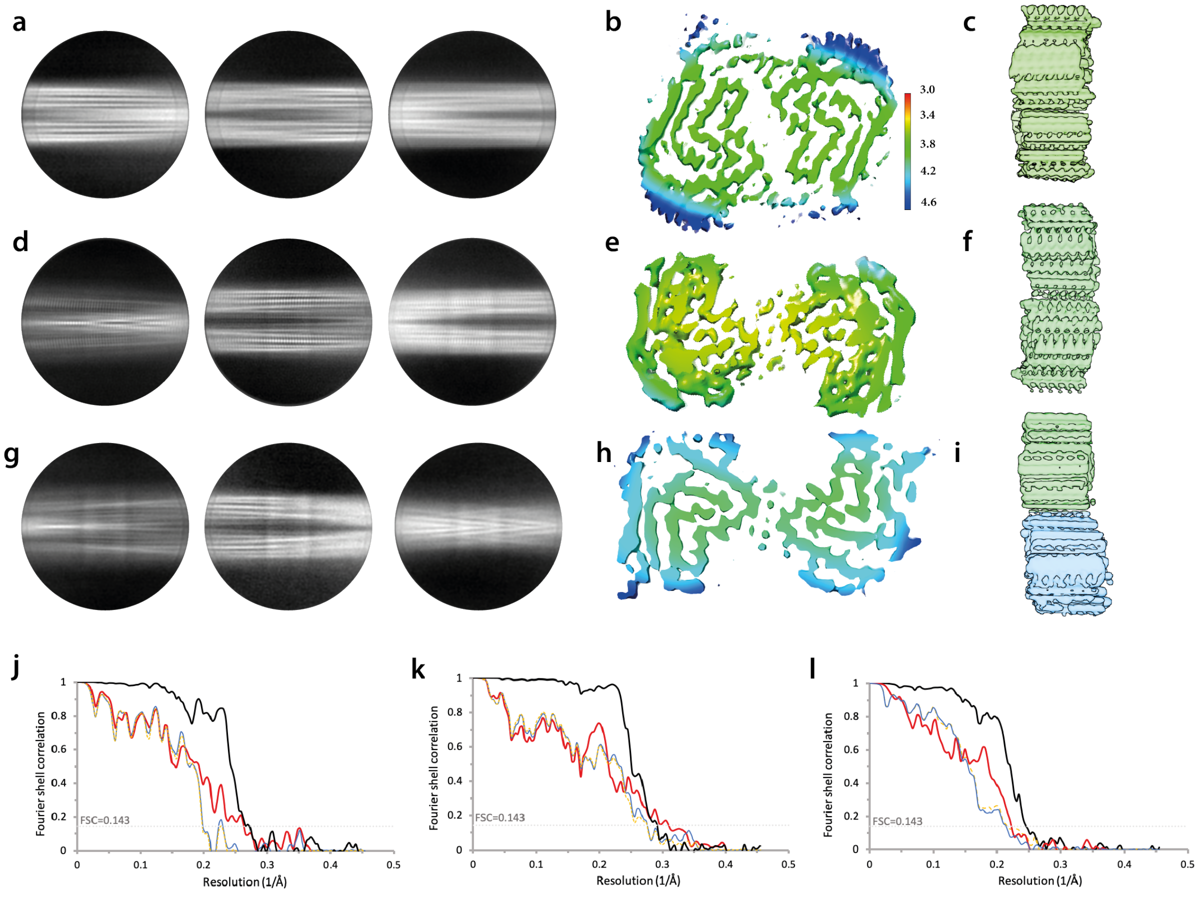

Supplementary Figure 5. Additional cryo-EM data on type 1 and type 2 filaments with protofilament fold B.

(a) 2D class averages of type 1 filaments with two protofilaments of fold B **(b)** Local resolution map for type 1 filaments with two protofilaments of fold B with the colour map indicating resolutions in Å. **(c)** Side view of the 3D reconstructions of type 1 filaments with two protofilaments of fold B. **(d-f)** as (a-c) but for type 2 filaments with two protofilaments of fold B. **(g-i)** as (a-c) but for type 2 filaments with one protofilament of fold A and one protofilament of fold B. **(j-l)** Fourier shell correlation curves for type 1 filaments with two protofilaments of fold B **(j)**, type 2 filaments with two protofilaments of fold B **(k)** and type 2 filaments with one protofilament of fold A and one protofilament of fold B **(l)**. Fourier shell correlation curves are shown between two independently refined half-maps (black) of the final cryo-EM reconstruction and refined atomic model (red), of the first half map and the atomic model refined against the first half map (blue), and of the atomic model that was refined against the first half-map against the second half-map (yellow dashed).

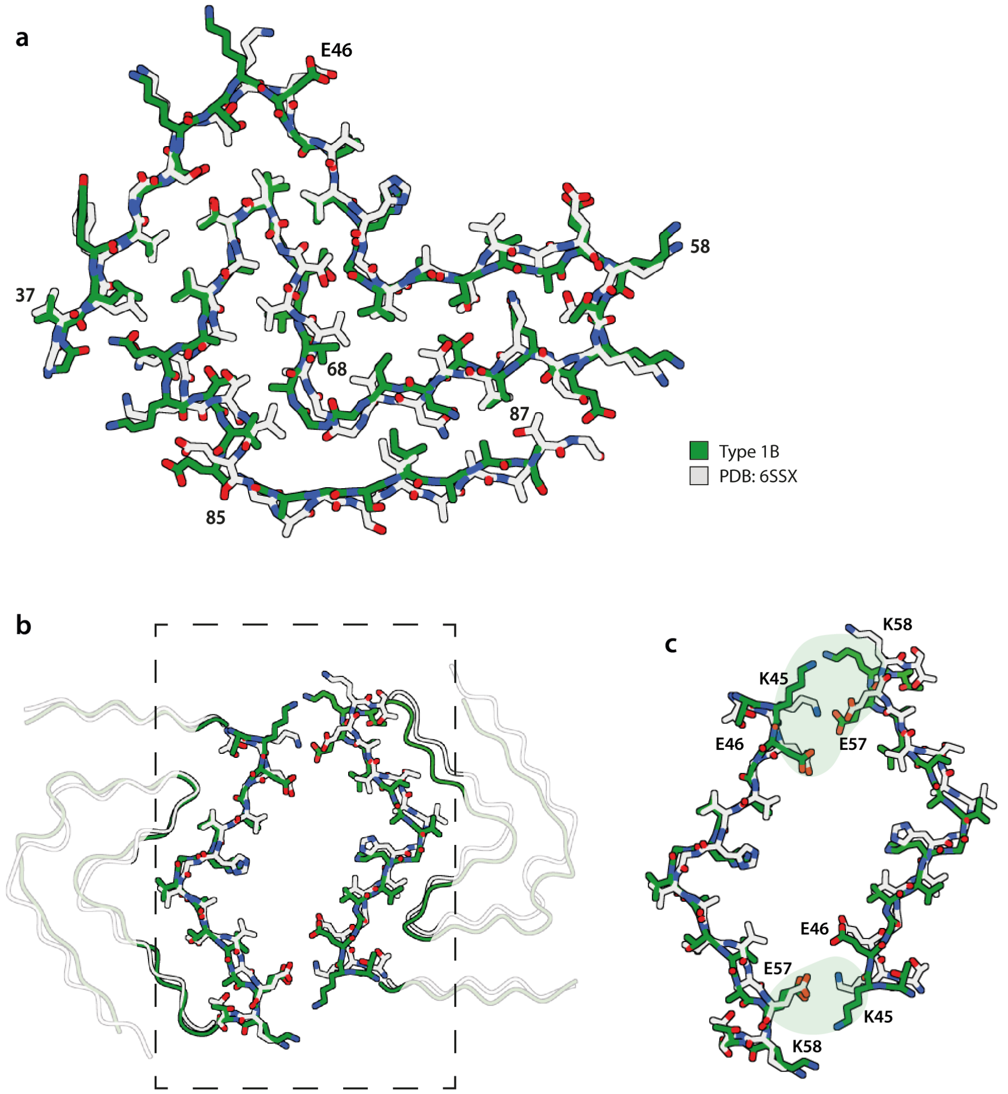

Supplementary Figure 6. Comparison of protofilament A with PDB-entry 6SSX of recombinant wild-type α-synuclein.

(a) Atomic model of protofilament fold A (blue) overlaid with one protofilament from PDB-entry 6SSX (grey). **(b)** Comparison of the interface between two protofilaments with fold A in type 1 filaments and those from PDB entry 6UFR, with the same colour scheme as in (a). **(c)** Zoomed-in view of the interface, with salt bridges between K45 and E47 in PDB-entry 6UPR and between E46 and K58 in type 1 filaments highlighted in grey and blue, respectively.

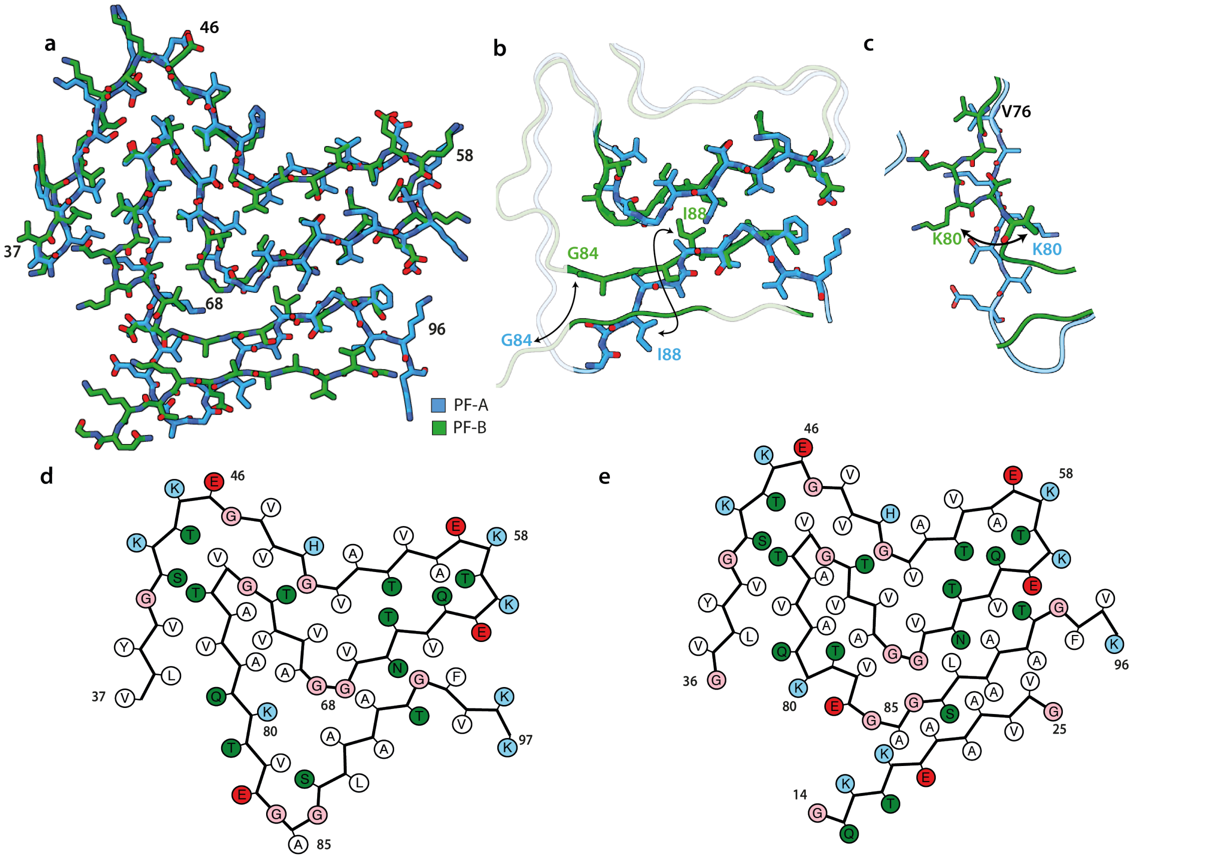

Supplementary Figure 7. Comparison of protofilament folds A and B.

(a) Atomic model of protofilament fold A (blue) overlaid with protofilament fold B (green) **(b,c)** As in **(a)**, but showing all-atom representation for different residues. **(d,e)** Schematic representations of protofilament folds A and B. Each amino acid residue is represented with its one-letter code in a circle. Positively charged amino acids are shown in blue, negatively charged ones in red, polar ones in green, hydrophobic ones in white, and glycines in pink.

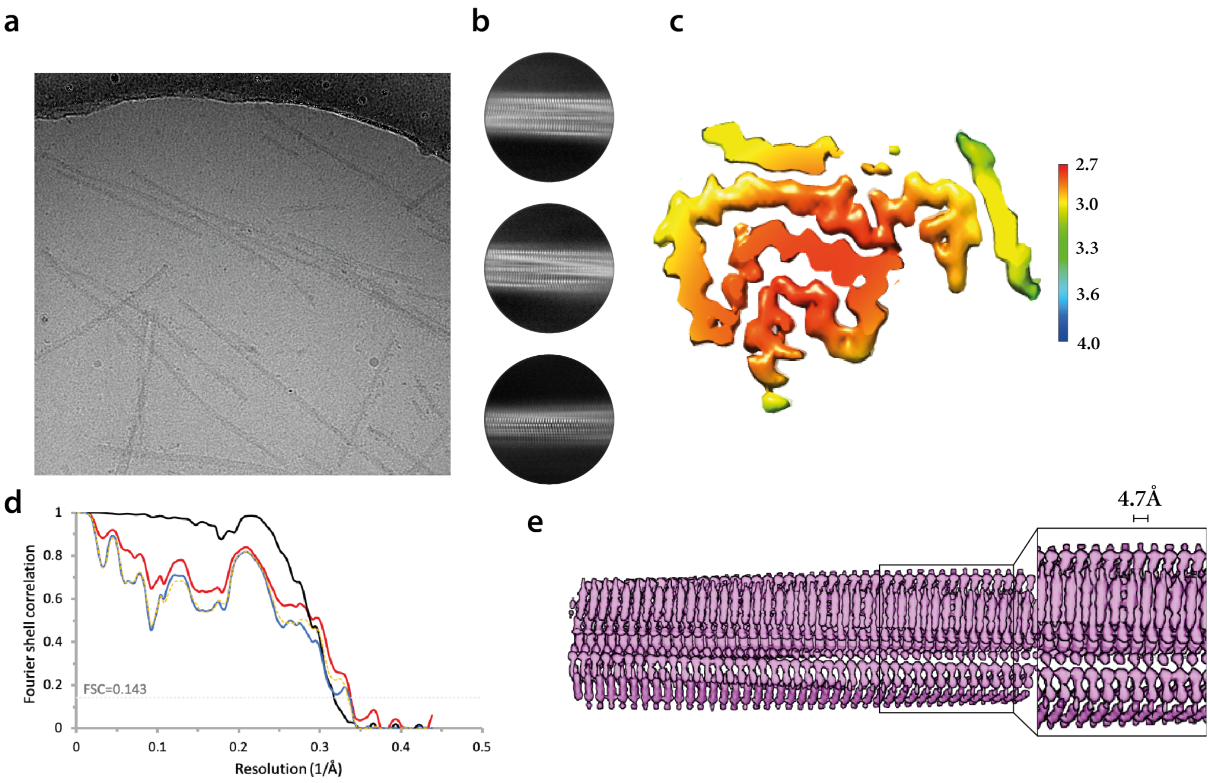

Supplementary Figure 8. Additional cryo-EM data on type 3 filaments.

(a) Electron micrograph of case 5. The scale bar indicates 50 nm. **(b)** 2D class averages of type 3 filaments in a box spanning 280 Å. **(c)** Local resolution map, with the colour map indicating resolutions in Å. **(d)** Fourier shell correlation curves between two independently refined half-maps (black), of the final cryo-EM reconstruction and the refined atomic model (red), of the first half map and the atomic model refined against the first half map (blue), and of the atomic model that was refined against the first half-map against the second half-map (yellow dashed). **(e)** Side view of the 3D reconstruction, showing separation of β-strands along the helical axis.

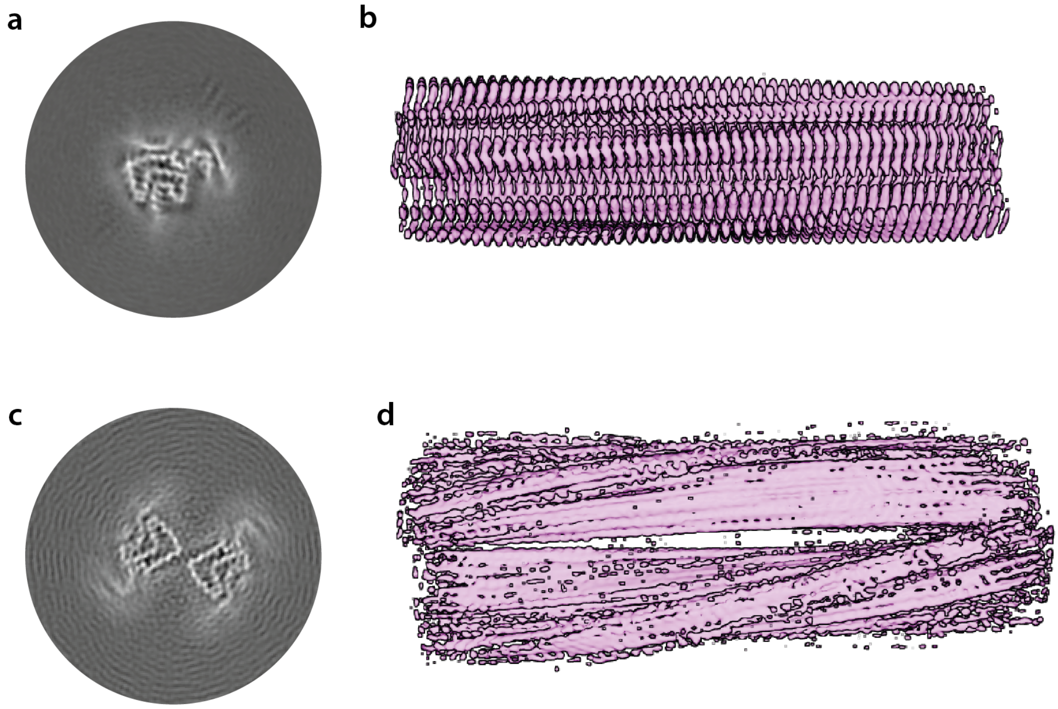

Supplementary Figure 9. Second-generation type 3 filaments.

(a) Central slice of the 3D map of the type 3 filaments from the second generation of seeding. **(b)** Side view of the 3D reconstruction of the same type 3 filaments. **(c-d)** As in (a-b), but for the doublets of type 3 filaments.

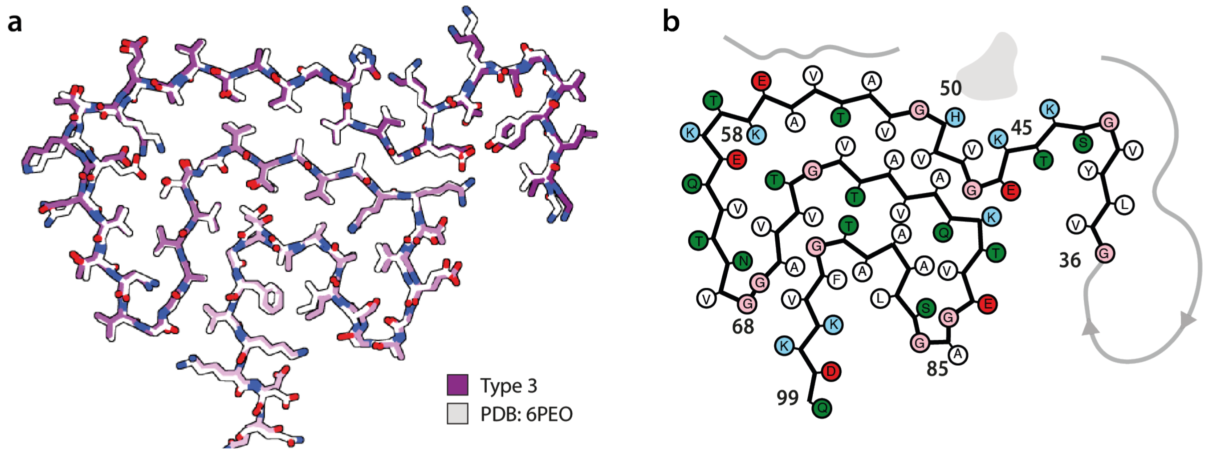

Supplementary Figure 10. Comparison of type 3 filament with PDB entry 6PEO of assembled recombinant H50Q α-synuclein.

(a), All-atom view of the type 3 filament (purple) aligned with PDB-entry 6PEO (grey). **(b)**, Schematic representation of the type 3 filament.
